# Seroprevalence of SARS‐CoV‐2 antibodies and associated risk factors during the second wave of infection in a university community in Cameroon

**DOI:** 10.1111/irv.13222

**Published:** 2023-11-12

**Authors:** René Ghislain Essomba, Albert Ngano Bayibeki, Abel Lissom, Pulchérie Thérèse Ateba, Nassif Seni, Claude Ariane Nlozoa Fouda, Solange Berthe Diwandja Mbitock, Normand David Ebonda, Sylvie Delphine Afana, Siméon Akame, Adelphe Anyang Tembu, Modeste Romuald Ngamaleu, Bernard Christel Bihonba Bimai, Ousmane Kabo, Philipe Salomon Nguwoh, Christian Taheu Ngounouh, Moise Christian Junior Meka, Michel Kengne, Julienne‐Louise Ngo Likeng, Etienne Omolomo Kimessoukie, Benjamin Alexandre Nkoum, Marie Claire Okomo Assoumou, Joseph Fokam, I. I. Yap Boum, Myriam Sylvie Ambomo, Michael Junior Piameu Chadou

**Affiliations:** ^1^ National Public Health Laboratory (NPHL) Ministry of Public Health Yaoundé Cameroon; ^2^ Faculty of Medicine and Biomedical Sciences (FMBS) University of Yaoundé I Yaoundé Cameroon; ^3^ School of Health Sciences Catholic University of Central Africa (ESS‐UCAC) Yaoundé Cameroon; ^4^ National Public Health Emergency Operations Coordination Centre (NPHEOCC) Ministry of Public Health Yaoundé Cameroon; ^5^ Department of Zoology, Faculty of Sciences University of Bamenda (UBa) Bamenda Cameroon; ^6^ Higher Institute of Sciences and Techniques Applied to Health, Faculty of Medicine and Pharmaceutical Sciences University of Douala Yaoundé Cameroon; ^7^ Laboratory of Virology Chantal BIYA International Reference Center for Research HIV/AIDS Prevention and Management (CIRCB) Yaoundé Cameroon; ^8^ Faculty of Health Sciences University of Buea (UB) Buea Cameroon; ^9^ Pasteur Institute Bangui (IPB) Bangui Central African Republic

**Keywords:** Cameroon, COVID‐19, rapid diagnostic test, SARS‐CoV‐2, seroprevalence

## Abstract

**Background:**

The COVID‐19 pandemic has caused a public health emergency in all sectors of society, including universities and other academic institutions in Cameroon. However, little is known concerning the real prevalence of severe acute respiratory syndrome coronavirus 2 (SARS‐CoV‐2) infections among student communities during the second wave of infection in Cameroon. This study aimed to estimate SARS‐CoV‐2 antibodies seroprevalence among participants in a university community in Cameroon.

**Methodology:**

A cross‐sectional study was conducted from March to April 2021 in 547 students aged ≥18 years during a mass diagnostic campaign at the School of Health Sciences of the Catholic University of Central Africa (ESS/UCAC). The anti‐SARS‐CoV‐2 antibody screening was done using the Panbio™ COVID‐19 IgG/IgM Rapid Diagnostic Test.

**Results:**

The overall seroprevalence of SARS‐CoV‐2 antibodies was 27%, of which 89.9% (*n =* 133) was IgG, 6.7% (*n =* 10) IgM and 3.4% (*n =* 5) IgG/IgM positive. The undergraduate students represented 79% (432/547) of the total population and were highly positive with anti‐SARS‐CoV‐2 antibodies 30% (130/432) as compared with postgraduate students 20% (23/115). The total antibody seropositivity was higher in males (34.4%) than females (24.9%). Several factors were associated with an increased risk of SARS‐CoV‐2 seroprevalence including the male gender (OR: 1.61 [95% confidence interval, CI 1.0–2.4]), specialization to medical laboratory (OR: 2.8 [95% CI 1.1–7.1]) and nursing sciences (OR: 2.6 [95% CI 1.1–6.2]).

**Conclusion:**

Our findings point to extensive and underreported circulation of SARS‐CoV‐2 in a university community during the second wave of infection in Cameroon, which likely resulted in artificially low case counts.

## 1 INTRODUCTION

Coronavirus disease (COVID‐19), caused by the severe acute respiratory syndrome coronavirus 2 (SARS‐CoV‐2), was first reported in China at the end of 2019. The highly contagious virus quickly reached pandemic proportions, and by the end of February 2023, over 757 million cases of COVID‐19 had been reported worldwide.^1^

The first case of COVID‐19 in Cameroon was diagnosed on 6 March 2020. Cases started to rise rapidly country‐wide and by March 17, 2020. Cameroon has been under a strict public health alert during the first wave of the disease, with masking required in all public areas, closure of borders, closure of schools and universities, the banning of gatherings of more than 50 individuals and implementation of education and awareness campaigns.^2^ The second wave of the COVID‐19 pandemic caused by the SARS‐CoV‐2 started in Cameroon in the middle of February 2021 and ended in June 2021.^3^ During the second wave of the COVID‐19 pandemic, Cameroon recorded 52,271 confirmed cases and 835 deaths. The maximum number of deaths, that was about 50% of total reported COVID‐19 deaths in Cameroon, happened during the peak of this second wave between April and May 2021.^3^ However, the real number of SARS‐CoV‐2 infections during the second wave of infection in Cameroon is unknown probably due to both the likely large number of people with asymptomatic or mildly symptomatic infections as well as the lack of widespread testing.^4^ Young adults have the highest rates of SARS‐CoV‐2 infection but rarely develop severe COVID‐19 infection.^5^ Consequently, university students are expected to have mild transient illness if infected with SARS‐CoV‐2 and might be the potential reservoir of disease transmission during the pandemic outbreak in the localities. Given the close proximity of many university students living in high‐density neighborhood and their extensive connected social networks compared with the general population, the potential for rapid spread of SARS‐CoV‐2 in university settings is of concern. In this context, the use of serological antibody tests to detect past exposure to SARS‐CoV‐2 is valuable. Serological assays can detect evidence of SARS‐CoV‐2 infection from 2 weeks to several months after the onset of symptoms and can reveal past infection even in asymptomatic cases.^6^

In Cameroon, few seroprevalence studies have been conducted in university settings.^2^, ^7^ However, no published data estimate the seroprevalence in a university community during the second wave of infection in Cameroon. Thus, there was an urgent and important need to understand the status of SARS‐CoV‐2 infection during this complex period of the second wave of COVID‐19 pandemic in Cameroon and to establish baseline seroprevalence data to better understand population immunity levels. The purpose of this study conducted after the reopening of schools and universities in Cameroon was to determine the seroprevalence of SARS‐CoV‐2 antibodies in the student community of the School of Health Sciences of the Catholic University of Central Africa, and the factors associated with seropositivity.

## 2 MATERIALS AND METHODS

### 2.1 Study settings

The study was a descriptive cross‐sectional study that was carried out at the School of Health Sciences of the Catholic University of Central Africa (ESS/UCAC) located in Yaoundé in the Center region of Cameroon. It took place during a screening campaign against Covid‐19 organized from March to April 2021 within the campus well after the reopening of schools in Cameroon and in the middle of the second wave of the disease in the country.^8^ The study population was mainly composed of regularly enrolled students who voluntarily agreed to participate in the study. These included students in the Bachelor of Medical Laboratory and Nursing Sciences and the Master in Clinical Biology and Hospital Management. Students aged above 18 years who presented to any of the sites for voluntary screening were included in the study after obtaining informed consent.

### 2.2 Description of the study site

The School of Health Sciences is an institution of the Catholic University of Central Africa with four campuses, Messa, Nkongoa and Douala in Cameroon and Moundou in Chad Republic. The study was carried out on the Messa campus, in the Yaoundé 2 district, Mfoundi department, Center region. The school, created more than 60 years ago, offers a diversified range of training in the health sciences and has seen a gradual increase in the number of fields of study and students. During the academic year 2021–2022, the schools were counting approximately 2196 students with 630 males and 1566 females. No data describing the COVID‐19 epidemic (number of cases, hospitalization, death …) within the campus were available before this study, and the national mitigation policies recommended by the government were applied in all sectors of society including the School of Health Sciences. The study took place in the clinical biology application laboratory on the campus where all samples were collected and analyzed.

### 2.3 Data collection

Initial invitation to participate in the study was made via social media and administrative notes. There was no predefined participant recruitment as the sampling was based on who presented at the screening site and volunteered to participate in the study. The participants were informed about the objectives of the study and requested to freely sign a consent form before the recruitment in the study. A standardized questionnaire was administered to collect socio‐demographic data (e.g., age, sex, and location of residence). All recruited participants were not vaccinated.

### 2.4 Testing procedure

The Panbio™ COVID‐19 IgG/IgM Rapid Diagnostic Test manufactured by Abbott was used to screen for SARS‐CoV‐2 IgG and IgM antibodies in capillary blood collected from a finger prick following the manufacturer's instructions. Test results were classified into one of the five categories: negative result (absence of antibodies), IgM positive result (indicating recent infection within the first 7 to 10 days), IgG positive result (indicating that the body has been exposed to the virus in the past), IgM and IgG positive results (also indicating recent infection between 7 and 21 days) and invalid/inconclusive. All the invalid results due to the absence of the control line and the doubtful results were repeated and classified either positive or negative to SARS‐CoV‐2 IgG and IgM. The manufacturer rates the sensitivity and specificity of the diagnostic test according to the specimen used including the finger stick whole blood (96.2% and 100%, respectively), venous whole blood (96.0% and 95.8%, respectively) and the plasma/serum (97.8% and 92.8%, respectively).^9^

### 2.5 Statistical analysis

Data management and tabulation were carried out using Microsoft Excel 2010. All statistical analyses were done using SPSS version 22.1. The normality of data distribution was checked using the Shapiro–Wilk test.^10^ Variables were expressed as proportions for categorical variables or median (with range)/mean (standard deviation) for continuous variables. The strength of association between each of the potential risk factors and the occurrence of SARS‐CoV‐2 antibodies was calculated using univariate logistic regression using the complete cases. The odds ratios (ORs) are given with a 95% confidence interval (CI). The statistical significance threshold for the tests was set at 5%.

## 3 RESULTS

### 3.1 Description of the study population

Out of 547 students enrolled in the study, 77.7% (425) were females and 79% (432) were at the undergraduate as reported in Table 1. The nursing students were the predominant specialty both in undergraduate (68.3%, *n =* 295/432) and postgraduate levels (49.6%, 57/115). The median age of participants was 24 years (IQR: 18–46), with 72.8% (*n =* 398) of student aged between 21–30 years. A total of 120 participants (21.9%) reported living in neighborhood belonging to Djoungolo health district, followed by Nkolndongo health district 21.8% (*n =* 119) and Efoulan and Biyem‐assi health district 20.7% (*n =* 113), respectively.

**TABLE 1:** Characteristics of the study population.

	Undergraduate (*n =* 432)	Postgraduate (*n =* 115)	Total (*n =* 547)
Sex % (*n*)			
Male	65.6 (80)	34.4 (42)	22.3 (122)
Female	82.8 (352)	17.2 (73)	77.7 (425)
Age (year)	23 (18.‐42)	28 (19–46)	24 (18–46)
Age group (year) % (*n*)			
18–20	97.4 (75)	2.6 (2)	14.0 (77)
21–30	80.7 (321)	19.3 (77)	72.8 (398)
31–46	76.0 (36)	76.0 (36)	13.2 (72)
Marital status % (*n*)			
Single	81.9 (417)	18.1 (92)	93.1 (509)
Married	39.5 (15)	60.5 (23)	6.9 (38)
Health districts % (*n*)			
Biyem‐Assi	69.9 (79)	30.1 (34)	20.7 (113)
Cité‐verte	90.0 (36)	10.0 (4)	7.3 (40)
Djoungolo	84.2 (101)	15.8 (19)	21.9 (120)
Efoulan	69.0 (78)	31.0 (35)	20.7 (113)
Nkolbisson	85.7 (36)	14.3 (6)	7.7 (42)
Nkoldongo	85.7 (102)	14.3 (17)	21.8 (119)
Academic specialties % (*n*)			
Nursing	68.3 (295)	49.6 (57)	64.4 (352)
Clinical biology		40.0 (46)	8.4 (46)
Medical Analysis	31.7 (137)	1.7 (2)	25.4 (139)
Health management		8.7 (10)	1.8 (10)

*Note*: Ages of groups expressed as median (min‐max).

### 3.2 Seroprevalence of SARS‐CoV‐2 IgG and IgM antibodies

**FIGURE 1:**
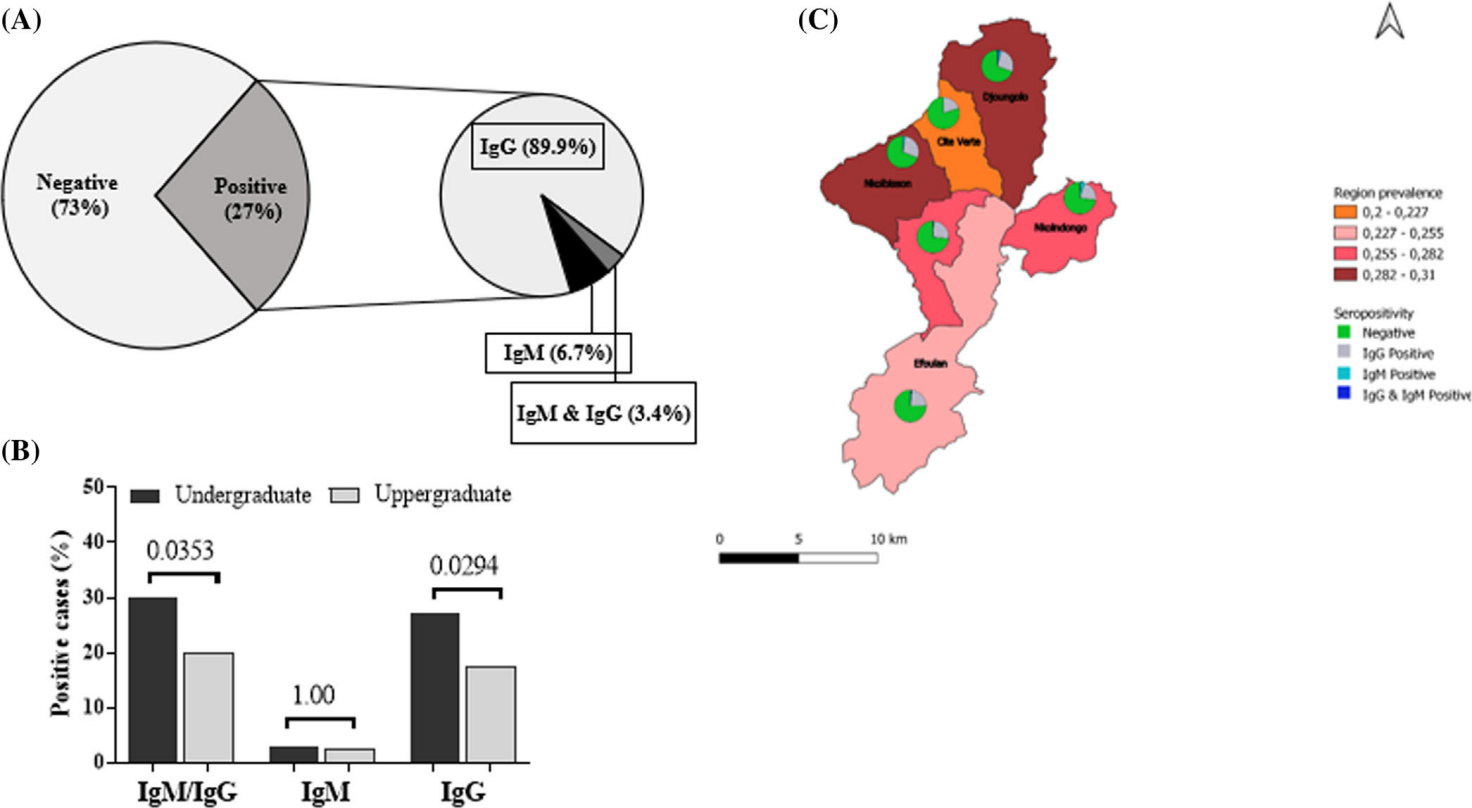
Seroprevalence of severe acute respiratory syndrome coronavirus 2 (SARS‐CoV‐2) IgG and IgM antibodies in the study population. (A) Global distribution of IgG and IgM antibodies in the population. (B) Distribution of SARS‐CoV‐2 IgM/IgG antibodies considering the training level of the students. (C) Geographic variation of the seroprevalence of SARS‐CoV‐2 IgM and IgM antibodies.

The overall seroprevalence of SARS‐CoV‐2 IgG/IgM antibodies among participants was 27% (148). Among participants tested positive for antibodies, 89.9% (*n =* 133) were IgG positive, 6.7% (*n =* 10) IgM positive and 3.4% (*n =* 5) were positive with IgG and IgM antibodies (Figure 1A). The seroprevalence was higher (*p =* 0.037) in males (34.4.1%; 42/122) compared with females (24.9%; 106/425 (Table 2). The seroprevalence was higher in the age group of 31–46 years 30.6%^11^ and ranged from 27.3% (95% CI = 0.6–1.8) among participants 18–20 years, to 26.4% among those of the age group 21–30. Further, undergraduate students had higher seropositivity for IgG (*p =* 0.0294) as well as both IgG and IgM antibodies (*p =* 0.0353) compared with postgraduate students (Figure 1B). Seroprevalence did not significantly differed between the health district (HD) (*p =* 0.77), although the higher level was recorded in Nkolbisson HD (31.0%, *n =* 13) and Djoungolo HD (30.8%, *n =* 37) (Figure 1C).

**TABLE 2:** Factors associated to exposure to SARS‐CoV‐2 infection of students.

	SARS‐CoV‐2 exposed	OR (95%CI)	*P*‐value
Sex % (*n*)			
Male (*n =* 122)	34.4 (42)	1.6 (1.0–2.4)	0.0386
Female (*n =* 425)	24.9 (106)	Ref	‐
Age group (year) % (*n*)			
18–20 (*n =* 77)	27.3 (21)	1.1 (0.6–1.8)	0.8713
21–30 (*n =* 398)	26.4 (105)	Ref	‐
31–46 (*n =* 72)	30.6 (22)	1.2 (0.7–2.1)	0.4636
Marital status % (*n*)			
Single (*n =* 509)	26.9 (137)	Ref	‐
Married (*n =* 38)	28.9 (11)	1.1 (0.5–2.2)	0.8612
Health districts % (*n*)			
Biyem‐Assi (*n =* 113)	26.5 (30)	1.4 (0.6–3.4)	0.4117
Cité‐verte (*n =* 40)	20.0 (8)	Ref	‐
Djoungolo (*n =* 120)	30.8 (37)	1.8 (0.7–4.2)	0.1906
Efoulan (*n =* 113)	24.8 (28)	1.3 (0.5–3.2)	0.5411
Nkolbisson (*n =* 42)	31.0 (13)	1.8 (0.7–4.9)	0.259
Nkoldongo (*n =* 119)	26.9 (32)	1.5 (0.6–3.5)	0.3867
Academic specialties % (*n*)			
Nursing (*n =* 352)	27.8 (98)	2.6 (1.1–6.2)	0.0373
Clinical biology (*n =* 46)	13 (6)	Ref	
Medical Analysis (*n =* 139)	29.5 (41)	2.8 (1.1–7.1)	0.0311
Health management (*n =* 10)	30 (3)	2.9 (0.6–14.2)	0.1989

Abbreviations: CI, confidence interval; OR, odd ratio; Ref, reference; SARS‐CoV‐2, severe acute respiratory syndrome coronavirus 2.

### 3.3 Relationship between seroprevalence of SARS‐CoV‐2 IgM/IgG antibodies response and sociodemographic parameters

As shown in Table 3, the multivariable risk factor analysis for IgM seropositivity revealed a significant higher odd of seropositivity for males (OR: 122.7 [95% CI 7.3–2067.1]) *P* < 0.0008. Seroprevalence did not differ by age, marital status, residential setting (health district) or academic specialties. The highest stratified seroprevalence was seen in students enrolled in medical laboratory studies, and 37.6% (38/139) of these students were IgG positive. The students with residence within the Nkoldongo health district had higher odds of having IgM antibodies, signs of a recent infection, but the odds ratio was not significant (OR: 4.4 [95% CI 0.2–80.1] (*p*‐value = 0.3159).

**TABLE 3:** Factors associated to seroprevalence of SARS‐CoV‐2 IgM/IgG antibodies.

	IgM‐positive	Multivariable OR (95%CI)	*P*‐value	IgG‐positive	Multivariable OR (95%CI)	*P*‐value
Sex % (*n*)						
Male (*n =* 122)	12.3 (15)	122.7 (7.3–2067.1)	0.0008	26.2 (32)	1.1 (0.7–1.7)	0.7728
Female (*n =* 425)	0(0)	Ref	‐	24.9 (106)	Ref	‐
Age groups (Year) % (*n*)						
18–20 (*n =* 77)	1.3 (1)	Ref	‐	26.0 (20)	1.1 (0.6–1.8)	0.8709
21–30 (*n =* 398)	3.3 (13)	2.6 (0.3–19.9)	0.9452	24.1 (96)	Ref	‐
31–46 (*n =* 72)	1.4 (1)	1.1 (0.1–17.4)		30.6 (22)	1.4 (0.8–2.4)	0.2480
Marital status % (*n*)						
Single (*n =* 509)	2.9 (15)	2.4 (0.1–41.1)	0.5425	25.0 (127)	Ref	‐
Married (*n =* 38)	0 (0)	Ref	‐	29.0 (11)	1.2 (0.6–2.5)	0.5848
Health districts % (*n*)						
Biyem‐Assi (*n =* 113)	0.9 (1)	1.1 (0.0.4–26.8)	0.9669	26.5 (30)	1.4 (0.6–3.5)	0.4117
Cité‐verte (*n =* 40)	0 (0)	Ref		20.0 (8)	Ref	
Djoungolo (*n =* 120)	2.5 (3)	3.9 (0.2–71.3)	0.3644	28.3 (34)	1.6 (0.7–3.8)	0.3022
Efoulan (*n =* 113)	1.8 (2)	1.8 (0.1–38.6)	0.7021	23.9 (27)	1.3 (0.5–3.1)	0.6148
Nkolbisson (*n =* 42)	2.4 (1)	2.8 (0.1–70.4)	0.5346	28.6 (12)	1.6 (0.6–4.4)	0.3683
Nkoldongo (*n =* 119)	5.0 (6)	4.4 (0.2–80.1)	0.3159	22.7 (27)	1.2 (0.5–2.8)	0.7227
Academic specialties						
Nursing (*n =* 352)	3.1 (11)	3.1 (0.2–54.0)	0.4321	25.9 (91)	2.3 (1.0–5.7)	0.0634
Clinical biology (*n =* 46)	0 (0)	Ref		13 (6)	Ref	
Medical analysis (*n =* 139)	2.9 (4)	3.1 (0.2–58.5)	0.4523	37.6 (38)	2.5 (1.0–6.4)	0.0541
Health management (*n =* 10)	0 (0)	4.4 (0.1–236.2)	0.4633	30 (3)	2.9 (0.6–14.2)	0.1989

Abbreviations: CI, confidence interval; OR, odd ratio; Ref, reference; SARS‐CoV‐2, severe acute respiratory syndrome coronavirus 2.

## 4 DISCUSSION

Data on SARS‐CoV‐2 antibody prevalence among the university population are limited. Here we present one of the reports of seroprevalence in a student community residing in urban setting during the second wave of the pandemic in Cameroon. These results help to fill the knowledge gap concerning the magnitude of the COVID‐19 epidemic during the peak of the second wave of the pandemic in 2021.

Overall, we found that an estimated 27% of university student population aged ≥18 years were SARS‐CoV‐2 antibodies positive by the end of April 2021. The seroprevalence was higher in males and did not differ with age, marital status, residential setting (health district). This might be justified by the fact that young males are more socially actives than females, visiting crowded areas like bars and practicing contact games like football which will increase the risk of SARS‐CoV‐2 transmission. The anti‐SARS‐CoV‐2 antibodies prevalence in university student population found in this study was higher compared with what reported by Voundi et al.^7^ (4.6%) among a student community in Yaoundé but less than the one obtained by Wondeu et al.^2^ among staff and student in a Cameroon university in Bandjoun (75.5%). This difference could be justified in one hand by sample size of our study (547) as compared with that of Voundi et al. (11549) and Wondeu et al. (106). In another hand, the study done at the Yaoundé I University (Centre region) was realized from October to December 2020, a period characterized by the end of the first wave of the pandemic, and the second study done at the university community in Bandjoun (West region) was realized during the period of December 2021 to February 2022, far away after the second wave of the infection. This illustrates the high heterogenicity of SARS‐CoV‐2 seroprevalence depending on region and survey period.

Similar variations have been observed in others seroprevalence studies in general population in Cameroon, depending on the population assessed and the implementation period. Our results were also similar to those reported by a large cross‐sectional survey conducted in households in an urban health district in Yaoundé and found an overall prevalence of 29.2%,^12^ which was higher than that found in a population of health care workers in three health facility of Yaoundé (23.6%).^13^ Our seroprevalence is lower than that reported by Fai et al. (32%)^14^ conducted in COVID‐19 testing sites across the Center region of Cameroon between June and August 2020 and the study conducted by Ndongo et al.^15^ in two independent population‐based SARS‐CoV‐2 serosurveys in Yaoundé, Cameroon, during January 27 to February 6 (18.6%) and April 24 to May 19, 2021 (51.3%). The difference in seroprevalence can be explained by multiple factors such as differences in population heterogeneity, demographics, urbanity, study design and timing of sample collection relative to symptom and epidemic onset.^14^ At the time of the study, our finding of 27% seroprevalence is consistent with the increase in the number of COVID‐19 cases reported across the country during the second wave of infection and estimated to 52,271 confirmed cases.^8^, ^16^ Our results implies that around 540 of the university's 2000 students registered at the School of Health Sciences of the Catholic University of Central Africa had been infected with SARS‐CoV‐2. This finding illustrates high community transmission during the second wave of COVID‐19.

We found that 89.9% of participants were reactive to the IgG antibodies, possibly suggesting previous SARS‐CoV‐2 infection. This result is consistent with the fact that the maximum number of cases linked to the SARS‐CoV‐2 infection was reported during the second wave of the infection in Cameroon. The ability of the COVID‐19 to spread asymptomatically means that the official reported number of cases to health reporting systems globally did not account for all the possible infections. This underscored the need of seroprevalence studies which could provide a full picture of the disease burden in a population by measuring SARS‐CoV‐2 antibodies in sampled blood specimens to detect previous infection, regardless of the presence or absence of symptoms. For this study, we used the Panbio™ COVID‐19 IgG/IgM Rapid Diagnostic Test manufactured by Abbott which detects N‐antibodies within 7–14 days infection, has a high sensitivity and specificity and detects antibodies earlier than other antibody assays, particularly those that measure SARS‐CoV‐2 spike protein antibodies which take longer to develop after infection.^17^

We found that men were significantly more likely to be seropositive, and we also observed higher seropositivity, although nonsignificant, among age groups 31–46 years. Nwosu and collaborators also found that male had significantly higher odds of seropositivity.^12^ Men are known to have longer lasting antibody responses,^18^ and early studies have already indicated that males have a higher susceptibility to COVID‐19 infection^19^ and tend to experience higher severity and fatality of COVID‐19 cases.^20^, ^21^ This sensitivity linked to the gender can be explained by multiple facts such as the gender difference in the distribution of ACE 2 which is the site of virus entry inside the target cell. It has been proven that ACE 2 is highly expressed and active in males than females, thus contributing to sex differences in COVID‐19 infection.^22^

The present study found a significant association between SARS‐COV‐2 infection and the academic level of the students, with the highest risk found in undergraduate students who exhibited more IgG and combined IgM and IgG positive cases than postgraduate students, as also observed in studies conducted in England,^5^ Ethiopia,^23^ and Los Angeles.^11^ Interestingly, seropositivity was associated with residential settings. Seroprevalence was high in students living in health districts of Nkolbisson 31% (2.4% of IgM and 28.6% of IgG) and Djoungolo 30.8% (2.5% of IgM and 28.3% of IgG). Potential reasons for these findings might be related to the student living conditions, less follow‐up of preventive public health measures and more importantly the fact that most of the participants, as health science students, have completed internships in different hospital environment since December 2020. This undoubtedly increased the circulation of the virus among these students, since it is well‐known that the COVID‐19 prevalence was high among healthcare workers in Cameroon.^13^, ^24^

Some limitations of this study include the lack of data related to participant having experienced COVID‐like symptoms or had a previous PCR confirmation, household composition and comorbidities. Second, the diagnostic accuracy of the rapid diagnostic tests used in this study is variable; this may be less sensitive than enzyme‐linked immunosorbent assays to detect SARS‐CoV‐2 antibodies. Also, seroconversion varies widely in persons with SARS‐CoV‐2 infection, with some people failing to mount a detectable humoral response while others show an overt response, which can lead to underestimation of seroprevalence. However, this study may be considered in‐formative as screening strategy to apply in context at risk.

## 5 CONCLUSION

The seroprevalence of the SARS‐CoV‐2 infection in this university student community in Yaoundé has reached exceptionally high levels during the second wave of the coronavirus pandemic. Our findings point to extensive and underreported circulation of SARS‐CoV‐2 in a university community during the second wave of infection in Cameroon. These findings highlight the importance of diagnostic testing and asymptomatic SARS‐CoV‐2 transmission among student community in Cameroon, which likely resulted in artificially low case counts.

## AUTHOR CONTRIBUTIONS

R. G. E. and M. J. P. C.: conceptualization; N. S., P. T. A., C. A. N. F., S. B. D. M., N. D. E., S. D. A., S. A., A. A. T., M. R. N., B. C. B. B., and OK: formal analysis and investigation; R. G. E., M. J. P. C., A. L., P. S. N., M. C. J. M., J. F., Y. B., and A. N. B.: data curation and methodology; E. R. G., N. S., A. L., M. J. P. C.: writing the original draft preparation; M. K., E. O. K., B. A. N., M. C. O. A., and M. S. A; resources, supervision and funding acquisition: R. G. E., J. L. N. L., M. J. P. C., N. S., J. F., Y. B., and A. N. B.: writing‐review and editing; all authors read and approved the final manuscript.

## CONFLICT OF INTEREST STATEMENT

The authors declare no conflict of interest.

### PEER REVIEW

The peer review history for this article is available at https://www.webofscience.com/api/gateway/wos/peer-review/10.1111/irv.13222.

## INSTITUTIONAL REVIEW BOARD STATEMENT

This study was conducted in accordance with the declaration of Helsinki, and approved by the Ethics Committee of the School of Health Sciences of the Catholic University of Central Africa (No. 2021/020250/CEIRSH/ESS/LAM).

## INFORMED CONSENT STATEMENT

Informed consent was obtained from all subjects involved in the study. Written informed consent has been obtained from the patient(s) to publish this paper.

## Data Availability

The datasets generated or analysed during the current study are not publicly available due to privacy concerns but are available from the corresponding author upon request.
